# Validation of the Helsinki University Hospital prevent pressure Injury Risk Assessment Tool: a prospective observational study

**DOI:** 10.1186/s12912-021-00799-6

**Published:** 2022-01-17

**Authors:** Anniina Heikkilä, Jaana Kotila, Kristiina Junttila

**Affiliations:** 1 Development Manager, HUS Group Administration, Nursing, Helsinki, Finland; 2 Helsinki University and Helsinki University Hospital, Helsinki, Finland; 3 HUS Group Administration, Nursing Helsinki University and Helsinki University Hospital, P.O. Box 705, FI-00029 Helsinki, Finland; 4 Clinical Nurse Specialist, HUS Neuro Center Administration, Nursing, Helsinki, Finland; 5 Director of HUS Nursing Research Center, Helsinki, Finland

**Keywords:** Pressure injury, Pressure ulcer, Risk assessment tool, Acute care, Inpatient

## Abstract

**Background:**

Pressure injures are a common adverse event in a hospital, and they are one of the most important quality indicators of patient care. Risk assessment is recommended as the first step in the prevention of pressure injuries. A Prevent Pressure Injury Risk Assessment Tool is a new tool for risk assessment that was developed by the Helsinki University Hospital.

**Aim:**

The aim of this study was to evaluate the predictive validity and the concurrent validity of the Prevent Pressure Injury Risk Assessment Tool in acute care.

**Method:**

The prospective observational study was conducted in 19 in-patient wards representing internal medicine, neurology, and surgery during 2017–2018. The participants’ inclusion criteria were: age ≥18 years old, no pressure injury on admission to the hospital and consenting to participate. The data collected by physical assessment of patients was combined with data from electronic patient records. Each patient was assessed by two different nurses with the Prevent Pressure Injury Risk Assessment Tool and the Braden Scale at patient admission. Furthermore, skin condition was observed throughout the hospital stay.

**Results:**

Of the 637 patients accepted for the study, 10 (1.6%) developed a pressure injury during the hospital stay. Poisson regression analysis showed that pressure injuries were more likely in high–risk patients compared to those with low-risk. The sensitivity of the Prevent Pressure Injury Risk Assessment Tool was adequate (75%), while specificity was poor (40%). A moderate correlation was found between the Prevent Pressure Injury Risk Assessment Tool and the Braden Scale.

**Conclusions:**

The Prevent Pressure Injury Risk Assessment Tool may be useful for identifying the adult pressure injury risk patients in acute care. Further research is needed to evaluate interrater reliability, and usability and validity with different patient populations.

**Supplementary Information:**

The online version contains supplementary material available at 10.1186/s12912-021-00799-6.

## Introduction

Pressure injures (PIs) are defined as localised injury to the skin and/or underlying tissue, usually over a bony prominence, as a result of pressure alone or in combination with shear [1]. In recent literature, the previously used term *pressure ulcer* (PU) has been replaced with pressure injury (PI), and PIs can be considered as health care quality indicators [1–3]. The PI risk is high in elderly people with impaired mobility because injuries commonly occur in patients with limited mobility and inadequate nutrition [2, 4], such as in patients who are treated for a longer time in hospitals [5]. In Finland, the number of older people who are over 80 years old is increasing, as it is in Western societies in general. This will increase the number of PIs in health care, thus more attention needs to be paid to their prevention in the future [5–7].

Hospital-acquired pressure injuries (HAPIs) are monitored because they are one of the most important indicators of care quality and cause significant mortality and additional costs [8]. Nurses play a key role in preventing PIs during hospitalisation. A better understanding of the underlying mechanisms may improve the quality of care [6]. In different studies, HAPIs have been documented to occur in between 11 and 20% of patients if all PIs are included (grades I–IV). PIs in the heel are the most frequent and most of PIs are of grade I [6, 9–12]. Only 27% of patients at PI risk received appropriate preventive interventions [13]. PIs are among the most common complications of all complications (28%) after traumatic spinal cord injuries [14]. In more recent studies, medical devices are also identified to be associated with the risk of developing PIs, including respiratory devices, cervical collars, tubing devices, splints and intravenous catheters. The incidence and prevalence of medical device–related PIs have been reported to be from 10 to 12%, respectively [3].

The prevalence and incidence of PIs in hospitals is still high [15], although more attention has been paid to their prevention in organisations in recent years. In the prevention of PIs, the first important step is to identify the risk patients [16, 17]. As a conclusion of a systematic review by Moore and Patton [18], strong evidence of the systematic use of structured PI risk assessment tools, such as the Braden Scale, to reduce the incidence, or severity of PIs, is missing.

A multidimensional process of nursing decision-making includes experience, education, understanding the patient’s status, nurses’ autonomous status, and cultural factors. Experienced nurses utilize a wide range of factors and processes in nursing decision-making [19]. Being in a hurry has been found to influence why the assessment of the PI risk is often left undone in nursing; this is why it should not take long to do the risk assessment [20]. Thus, risk assessment instruments should contain the most important risk factors for PIs and be easy to use [10]. A validated instrument and the patient’s clinical assessment are used to identify a patient at PI risk [1].

## Background

Various risk assessment instruments have been developed, including the Braden, Cubbin and Jackson, Norton, Ramstadius, and Waterlow scales, of which the Braden Scale has been most tested in a variety of care settings [15, 21]. It has been validated in several studies and has been found to be the best for identifying the patient’s PI risk [17] as well as being found to have good sensitivity and moderate specificity [21, 22] and moderate predictive validity [21]. However, the Braden Scale has not been identified to help nursing professionals in preventive work on PIs [17]. Also, some patients will have additional risk factors and comorbidities that are not measured by the Braden Scale, and therefore, nurses’ clinical judgement is also needed [23]. The Shape Risk Scale (SRS) is a new, simple, PI risk tool. It takes into consideration the body mass index, body shape, physical activity and mobility, consciousness and sensory perception, and body temperature. The SRS appears to assess risk better than the Braden Scale, in particular, patients with low and moderate risk are better identified [24].

Several PI risk factors have been identified, the most significant of which are immobility, skin status (including previous PIs), perfusion, older age, sensory perception, limitations, nutrition indicators, moisture, body temperature and general mental and health status. Perfusion includes e.g., diabetes, vascular disease, circulation, blood pressure, oedema, and smoking [1, 25, 26]. Major direct causal risk factor are activity and mobility limitations, perfusion, and skin status. Immobility is a direct condition for the development of a PI. Skin status and perfusion are less straight-forward because they cannot cause a PI without immobility. However, both have strong evidence of the development of PIs. Poor sensory perception, diabetes, moisture, low albumin, and poor nutrition are considered to be the main indirect factors as they affect the outcome by changing the direct causal factors [25].

 The Helsinki University Hospital (HUS) has developed a Prevent Pressure Injury (PPI) Risk Assessment Tool (later: PPI Tool) in 2013-2014 (the study lasted 4.5 months) by involving 23 units in acute care and primary health care and approximately 4000 patients in the development. The number of nursing staff in the research units was approximately 900. PI risk in the PPI Tool is based on the patients’ mobility/activity, general skin status and sensory perception. Half of the units started the study by using the Braden Scale and half of the units started the study by using the PPI Tool. In the middle of the study, the units changed the Tool/Scale used. The patient’s skin was assessed at the beginning and end of treatment and, if necessary, during treatment (e.g. length of hospital stay over 7 days). The study also monitored the extent to which preventive interventions were implemented (skin status assessment, use of support surface, repositioning, preventive skin care, nutrition care). Incidence of PIs was 1.5% while using the Braden Scale in PI risk assessment and 1.9% while using the PPI Tool. No statistical significance in incidence was observed between the PPI Tool and the Braden Scale. In addition, no statistical differences were observed between the implementation of the interventions when using the Tool or the Braden Scale. At the end of the study, the nursing staff of the study units participated in a survey evaluating the usability of the PPI Tool and the Braden Scale and recommending one of them for use in the unit. In acute care, nursing staff recommended the use of the PPI Tool because they found the tool to be easy and quick to use. Primary care staff recommended more the use of the Braden Scale. Based on the results, the organization decided to introduce the PPI Tool because it was fast to use and found to be effective. Based on the risk classification, the patients are categorised to have a low, moderate, or high risk of getting a PI. The PPI Tool is part of the Prevent PI Protocol, which includes brief clinical instructions for preventing PIs, thus it guides nurses’ clinical decision-making. The organization’s goal is to assess the PI risk for 80% of hospitalized patients [27, 28]. Validity of the PPI Tool has not been previously studied. The aim of present study was to evaluate the validity of the PPI Tool in acute care. The main objectives were to explore the predictive validity and the concurrent validity of the PPI Tool. The study addresses the following questions:

1. What is the predictive validity of the PPI Tool in acute care?
2. What is the concurrent validity of the PPI Tool in acute care when comparing it to the Braden Scale?
3. What patient characteristics are related to the PI risk that is assessed by the PPI Tool?

## Methods

### Design

This prospective observational study was conducted in southern Finland at a large university hospital. The acute care hospital system has approx. 3,000 hospital beds. Treatment is given to about 612,000 individual patients and every year 81,000 surgical procedures are performed in the hospital system. The average length of stay (LOS) is 4.0 days (in somatic care). The total number of personnel is 27,100, of which 14,600 represent nursing staff [29, 30].

### Setting and sample

Data for this study were collected from eight surgical in-patient wards (gastrointestinal surgery, orthopaedics and traumatology, cardiothoracic surgery, vascular surgery and plastic surgery), nine internal medicine in-patient wards (general, infectious diseases dermatology and cardiology) and two neurological in-patient wards. There were 449 beds in the study units. The study was launched in 2018 and completed in 2019. The data were collected for a total of nine months, varying from seven to 13 weeks per ward. The incidence of PIs and the systematic use of PI risk assessment were considered in the selection of the study units.

In studies of the validity of PI risk assessment scales, the sample sizes have varied from hundreds of patients to a few thousand patients [7, 24]. The incidence rate of PI in acute care is about 8% when including all grades [9]. Based on this information, the goal was to have 1,000 participants, a number which was estimated to have about 80 PIs in the data. These numbers were thought to be sufficient for a reliable analysis.

Inclusion criteria for the participants were: an age of 18 years or older and speaking Finnish or Swedish. An exclusion criterion was an existing PI. Written informed consent was obtained from all participants. If the patient was unable to give his or her consent (e.g. due to acute confusion), the informed consent was asked for from a relative. Personal identification information was removed from the data before the analysis. Ethical approval was obtained from the Helsinki University Hospital Ethical Review Board *(HUS/888/2018)*, and study permission was given by the hospital authorities (HUS, 3.5.2018 and HUS, 28.2.2019). Reporting of the study follows the Strengthening the Reporting of Observational Studies in Epidemiology (STROBE) guidelines (Supplementary File 1).

### Data collection

Two kinds of data were collected: observational data and register data from the electronic patient records (EPRs). The physical assessment data were collected with two different instruments: (1) the Braden Scale and (2) the PPI Tool. The Braden Scale was chosen for the study because it had been used in the PPI Tool development project described earlier in the text. In addition, the Barden Scale is the most widely used and studied PI risk assessment scale [31]. Two nurses did the assessment without knowing each other’s assessment results. The assessments were done during the same work shift so that the patient’s condition did not change between the assessments.

All participants’ PI risk was first assessed with the Braden Scale. The tool comprises six subscales that evaluate the patient’s sensory perception, skin exposure to moisture, activity level, mobility, nutritional status and the level of friction and shear. The sum of the subscale scores constitutes the patient’s Braden Scale score, which can range from 6 to 23, with lower scores indicting a higher risk of developing a PI [32].

Next, the participants’ PI risk was assessed with the PPI Tool. Instead of being a traditional scoring system, the tool takes into consideration the patient’s medical history (i.e., previous PIs) and current health status, based on physical assessment of skin condition, integrity and sensory perception, and the patient’s mobility. Based on the information, the patient is categorised in one of the following three risk classes: low, moderate and high risk of getting a PI. In the low-risk class, there are no limitations to the patient’s mobility and his or her skin is healthy. In the moderate-risk class, the patient has limited mobility (the patient is able to change position to some extent), a poor sensory perception or fragile skin. In the high-risk class, the patient’s mobility is very limited (the patient is unable to change position) or she or he has an existing PI.

The study units nominated at least two nurses as responsible for the study. The nurses were trained with a two-hour training session and provided with a study handbook. The training included general information about PIs (stages and staging of PIs, risk factors), and information on how to conduct a PI risk assessment with the PPI Tool and the Braden Scale, and on how to ask for patients’ informed consent. Next, the responsible nurses trained their own unit’s staff on data collection. Additional support was offered if needed.

When collecting the observational data, Nurse 1 assessed the patient’s PI risk with the Braden Scale and recorded the information on a paper-based form, including the name and the personal identity code of the patient and the identity of the unit. The ID was required for combining the physical assessment data with data from the EPR. Next, Nurse 2, without knowing the outcome of the assessment by Nurse 1, assessed the patient’s PI risk with the PPI Tool, and checked the patient’s skin condition. This information was recorded in the EPR in accordance with the organisation’s normal care process. The assessment was to be reperformed in the case of changes in the patient’s condition. The patient’s skin condition considering potential PIs was recorded according to the normal care process (i.e. at least at the beginning and at end of the hospital stay). The PIs were staged by using the quick guide for PI staging by the Finnish Wound Care Society, which follows the guidelines of international NPUAP/EPUAP pressure ulcer classification system [1].

The data collected from the EPRs included patients’ background information (personal identity code, age, gender, length, weight), and treatment information (main diagnosis/-es, reason of treatment). In addition, the data included HUS episode information (the treatment unit, specialty, the episode’s starting time, the means of arrival, discharge information [date and service] and nursing clinical observations with time stamps [detailing the number, grade and location of PIs].

### Statistical analyses

Descriptive statistics were calculated for the demographic data of the participants. Categorical variables were described using frequencies and percentages. The connections between the background or health characteristics of patients and the risk class of the PPI Tool were studied with cross-tabulation, the χ2 test and a logistic binary regression model. For regression analysis, the PI risk was classified into two categories, low-risk and high-risk classes, because there were only 10 observations in the high-risk class. Results were displayed as odds ratio (*OR*) with 95% confidence intervals (CI). In addition, a receiver operating characteristic (ROC) curve was used to identify the PPI Tool’s ability to predict the risk of PI. The validation of the predictive power (the relative risk [*RR*]) of the PPI Tool was calculated by using Poisson’s regression model. In addition, the goodness of the PPI Tool was evaluated by calculating the sensitivity and specificity.

The concurrent validity of the PPI Tool, compared with the Braden Scale, was explored by descriptive methods, comparison of means, and correlation. Analysis of variance (ANOVA) and the Tukey HSD (honestly significant difference) test were used to analyse the differences among group means in a sample. ANOVA calculates group mean values, but it does not tell which specific groups differ from each other. The Tukey test can be used to calculate group difference [33]. In the Tukey test analysis, the Braden Scale scores were averaged for each risk class in the PPI Tool, after which the significances of the differences in the categories’ averages were analysed. Spearman’s Rho was used to calculate a correlation between the Braden Scale (using scores) and the PPI Tool (using three risk classes). Spearman’s Rho ranges from 0 to 1 with larger values, showing a stronger association between variables. The data were statistically analysed using the IBM Statistical Package for Social Sciences (SPPS) 22 for Windows.

## Results

Patients who had not been assessed during the same shift using both the PPI Tool and the Braden Scale or whose skin condition was not assessed both at admission and at discharge were excluded from the data. In all, 637 (66%) of the enrolled 964 patients fulfilled the criteria for study.

The study population had a mean age of 62 years (SD ±16.3) and 53% were men. Most of the participants (75%) were of low PI risk (assessed by the PPI Tool), were admitted to a surgical unit (57%) and stayed at the hospital for, on average, 4.2 days. All in, 10 PIs in ten participants occurred in the study (1.6%), including all PI grades. The incidence in low PI risk patients was 1.3%, in moderate –risk patients it was 1.4% and in high–risk patients it was 20%. The patient demographics and χ2 test are presented in Table 1 by the risk classes of the PPI Tool.

**Table 1:** The background and health characteristics of patients according to the PPI Tool risk classes. Chi-square or Fisher’s exact tests were calculated between the classes of the PPI Tool and variables. *P <* 0.05 is significant

The PPI Tool risk class at the beginning of treatment *n (%)* or mean *(± SD)*	Low-risk, *n* = 479 (75.2)	Moderate- risk, *n* = 148 (23.2)	High-risk, *n* = 10 (1.6)	*p-*value	All, *n* = 637
Gender (*n* = 629)					
Male	263 (78.7)	66 (19.8)	5 (1.5)	<0.87	334 (53.1)
Female	210 (71.2)	80 (27.1)	5 (1.7)		295 (46.9)
Age (*n* = 629)					
< 40	66 (89.2)	8 (10.8)	0 (0)	< 0.01b)	80 (12.7)
41–65	199 (78.7)	52 (20.6)	2 (0.8)		247 (39.3)
66–80	173 (72.7)	58 (24.4)	7 (2.9)		238 (37.8)
> 81	35 (54.7)	28 (43.8)	1 (1.6)		64 (10.2)
Age mean (±SD)	59.9 (16.59)	67.5 (13.9)	70.7 (13.2)		61.8 (16.2)
Medical specialty (*n* = 637)					
Medical	153 (78.5)	40 (21.5)	2 (1.0)	<0.24	195 (30.6)
Surgical	266 (73.1)	93 (25.5)	5 (1.4)		364 (57.1)
Neurology	60 (76.9)	15 (19.2)	3 (3.8)		78 (12.3)
Mode of arrival (*n* = 629)					
Elective	184 (64.1)	99 (34.5)	4 (1.4)	< 0.01	287 (45.6)
Emergency care	227 (84.1)	39 (14.4)	4 (1.5)		270 (42.9)
Other	62 (86.1)	8 (11.1)	2 (2.8)		72 (11.5)
Length of stay in days (*n* = 629)					
< 1	137 (90.7)	14 (9.3)	0 (0)	< 0.01b)	151 (24.0)
1–4	205 (67.4)	93 (30.6)	6 (2.0)		304 (48.4)
5–7	74 (78.7)	19 (20.2)	1 (1.1)		94 (14.9)
> 7	57 (71.3)	20 (25.0)	3 (3.8)		80 (12.7)
Length of stay in days (gross)	4.0 (4.82)	4.7 (5.00)	7.0 (9.18)		4.18 (4.96)
Surgical procedure during hospitalization					
No operation	219 (80.2)	48 (17.6)	6 (2.2)	0.01	273 (43.0)
Operation	260 (71.8)	98 (27.1)	4 (1.1)		362 (57.0)
Hospital-acquired pressure injuries (HAPIs)					
HAPIs, all grades	6 (60.0)	2 (20.0)	2 (20.0)	<0.01	10 (1.6)
No HAPIs	473 (75.4)	146 (23.3)	8 (1.3)		627 (98.4)
Braden Scale scores (*n* = 637)					
No risk: ≥19	470 (77.7)	131 (21.7)	4 (0.7)	a)	605 (95.0)
Mild risk: 15–18	7 (33.3)	14 (66.7)	0 (0)		21 (3.3)
Moderate risk: 13–14	2 (28.6)	2 (28.6)	3 (42.9)		7 (1.1)
High risk: 10–12	0 (0)	1 (25.0)	3 (75.0)		4 (0.6)
Very high risk: ≤9	0 (0)	0 (0)	0 (0)		0

(a) 7 cells (58.3%) have an expected count of less than 5, (b) Fisher’s Exact tests

The multivariable regression analysis found associations between the high–risk class of the PPI Tool and female gender, length of stay (LOS), older age, having a surgical procedure during the hospital stay and elective admission to hospital. No statistical significance was found between the PPI Tool’s risk classes and medical specialties. The ROC curve showed that the logistic regression model achieved the ROC curve (AUC) of 0.79, (CI 95%, 0.745-0.827; *p* < 0.001) for predicting risk classes in PPI Tool. The sensitivity of the model was 59% and the specificity was 83%. Table 2 describes the results of multivariable regression analysis.

**Table 2:** Multivariable logistic regression (adjusted) results when the PPI Tool risk classes were divided into high-risk and low-risk classes, *n=627*

Variables	High–risk %	*p-*value	Odds ratio (*OR*)	95% confidence interval (CI)	
				**Lower**	**Upper**
Gender					
Male	27				
Female	41	0.04	1.198	1.04	1.97
Age					
< 40	12				
40–65	27	0.21	1.72	0.74	4.01
66–80	38	0.04	2.38	1.02	5.50
> 80	83	<0.01	5.89	2.26	15.21
Length of stay in days					
< 1	10				
1–4	48	<0.01	6.06	3.16	11.60
5–7	27	0.04	3.19	1.45	7.05
>7	40	<0.01	6.52	2.87	14.78
Did the patient have a surgical procedure during the hospital stay					
No	21				
Yes	38	0.01	1.96	1.12	3.44
Mode of arrival					
Emergency care	19				
Elective	56	<0.01	5.83	3.23	10.50
Other	16	<0.79	0.80	0.398	2.02

A Poisson regression was run to predict the PI in each risk class of the PPI Tool. A change of the risk class from the low–risk to high–risk class increased the probability of PI by a factor of *RR* 2.77 (95% CI, 1.17 to 4.37; *p* <0.001). There was no statistical significance between the low–risk and moderate–risk classes. Table 3 shows the results of the probability of PI by risk class. In addition, a sensitivity of 75% and a specificity of 40% were calculated for the PPI Tool. The ROC curve was not statistically significant for either Braden Scale or for PPI Tool due to the low number of PIs.

**Table 3:** The result of a Poisson regression analysis; the probability of getting a PI assessed by PPI Tool risk classes

*N* = 637	PI risk Classes	PIs (*n*)	Patients (*n*)	Relative risk (*RR*)	95% confidence interval (CI)	
					**Lower**	**Upper**
PPI Tool	High-risk	2	10	2.771	1.170	4.371
	Moderate-risk	2	148	1.210	-1.524	1.676
	Low-risk	6	479			

**Fig. 1:**
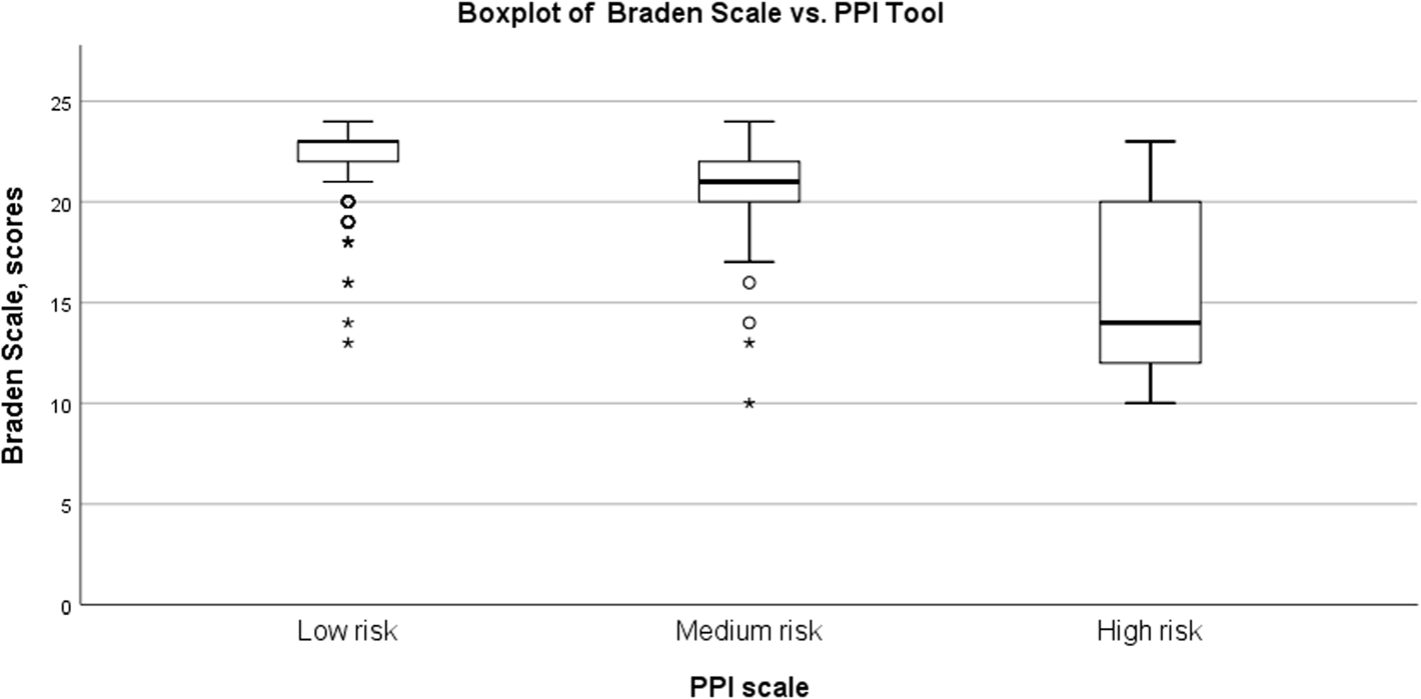
A box plot depicting the distribution of Braden Scale scores in PPI tool risk classes

Most patients (*n* = 479, 75%) were in the low–risk class when assessed by the PPI Tool. Of the 637 patients assessed by the Braden Scale, 98% were in the low–risk or no risk categories and the mean score was 22 (*SD* ± 2.0). There was a statistically significant difference between the PPI Tool’s risk classes by one-way ANOVA F (ANOVA F [2, 634] = 150.20; *p* < 0.001). Figure 1 shows a box plot depicting the distribution of Braden scores in PPI Tool’s risk classes. In addition, the Tukey HSD test was used to analyse the means of the PPI Tool classes. All risk classes were compared with each other and statistical significance between all classes was found. Table 4 describes a comparison of the means in the PPI Tool´s risk classes. Results of the Spearman correlation indicated that there was a moderate positive association between the Braden Scale and the PPI Tool (r(637) = 0.542, *p* < 0.001).

**Table 4:** The results of the Tukey HSD test, describing the mean scores of the Braden Scale in the PPI Tool risk classes

The PPI Tool and Barden Scale, category average differences comparison (*n* = 637)	Mean difference (I-J)	Std. error	Sig.	95% confidence interval	
				**Lower bound**	**Upper bound**
Low-risk vs. moderate-risk	1.832^*^	0.152	<0.001	1.48	2.19
Low-risk vs. high-risk	6.828^*^	0.516	<0.001	5.62	8.04
Moderate-risk vs. high-risk	4.996^*^	0.527	<0.001	3.76	6.23

* The mean difference is significant at the 0.05 level

## Discussion

This prospective observational study aimed at validating the new PI risk assessment tool, the PPI Tool, in acute care. The main objectives were to evaluate the predictive and the concurrent validity of the PPI Tool. The results show that the PPI Tool is able to predict PIs. When comparing the PPI Tool with the most studied PI risk assessment tool, the Braden Scale, a moderate correlation was found between the tools.

In this study, the HAPI rate was 1.6%, varying by the PPI Tool’s risk class. Compared to previous studies, the HAPI rate was low. In a study by Li et al. [9], the HAPI rate was 8.4% and 5.1% after grade I PIs were excluded. In a current extensive Finnish study in acute care (*n* = 5902), the HAPI rate was 10% including all grades and 3% including grades II to IV [12]. The low HAPI rate in this study can be explained by possible bias in participant recruitment. The enrollment required informed consent of the participant, and it may be that it was not always asked for from patients in critical condition, or their relatives. Thus, it may be that healthier patients with a lower PI risk were selected for the study.

Of the patients assessed with the PPI Tool, 1.6% were at high PI risk, and 0.6% of patients assessed with the Braden Scale were at high risk (mean score 22). In a study by Sardo et al. [34], 27% of 6552 inpatients were classified as having a high PI risk by the Braden Scale. Compared to the results of Sardo et al. [34] the difference is significant, and it can be suspected that a higher proportion of good condition patients were selected for the current study. This naturally affects the validity of the results and requires further research with specific attention to heterogeneous participant enrolment. However, it needs to be noted that both tools identified a very marginal number of high-risk patients. Comparison between studies is hampered by different types of study environment and the different cut points of the scales and study inclusion criteria (e.g., in the above-mentioned study by Sardo et al., patients treated for less than 24 h were excluded) [15].

In the multivariable regression analysis of the PPI Tool, it was found that various patients’ background variables were related to the increase in the PI risk level (e.g. female gender, older age, LOS, having an surgical procedure during treatment and the elective nature of services). The ROC curve accuracy was moderate, which is supported by the results of the analysis. There was no statistical significance between medical specialties. Other studies have mainly described the association of background variables with PI risk rather than the increase in PI risk. In several studies high age, surgical procedure during treatment and a longer LOS have been found to increase the risk of developing a PI. The risk posed by gender varies in different studies. However, some studies have found male gender to increase the risk to get a PI. Overall, there is little evidence that gender is associated with the PI risk [12, 15, 26, 35]. In this study, females had slightly increased risk to be at high PIs risk than males. It was analyzed (*X*^*2*^*)* that there were no statistically significant differences between genders in age, mode of arrival, mortality, intensive care, LOS, and BMI. Statistical significance was found between the surgical procedure and gender, with a bigger portion of women (57%) having surgical procedure than of men (52%). This probably explains the higher PI risk for women. The association of LOS with PI risk level may be partly explained by the fact that older patients in this study had longer care periods and age was associated with the PI risk. Additionally, elective patients had a significantly increased risk of having a high PI risk compared to emergency patients. The explanation is may be that elective patients had surgical procedure during hospitalization more often than emergency patients. In the PPI Tool, one factor that increases the PPI risk is the limited mobility, which is associated with almost all surgical procedures. Surgery-related PIs occur in 4 -45% of patients [1].

The PPI Tool was found to predict PIs. Analysis showed that PIs are more likely in high–risk patients compared to those with low-risk. It was not possible to compare moderate–risk to high-risk patients from the data because there were too few HAPIs. The sensitivity of the PPI Tool was adequate (75%) and specificity was poor (40%). The Braden Scale has received higher results in a meta-review by Huang al., (2021): pooled sensitivity was 0.78 (95% CI, 0.74–0.82) and pooled specificity was 0.70 (95% CI, 0.63–0.78). In this study, predictive ability was not calculated for the Braden Scale. This study specificity measures the PPI Tool’s ability to correctly generate a negative (low-risk) result for patient who does not have the PI risk. The data included six HAPI patients classified as having a low PI risk (1.3% of low-risk patients). Each of these patients had a surgical procedure during the hospital stay, four of which had lasted more than two hours. The LOS was more than a week for three patients and for two patients, it was 5-7 days. One patient died during the treatment period. Four of the patients were 66-80 years and two were 40-65 years. One explanation for the low PI risk level may be that the patients were mobile at admission and the planned surgery was not considered in the PI risk assessment as a factor increasing the PI risk. Neither of the assessment tools used in this study, the PPI Tool, or the Barden Scale, identifies a surgery as a PI risk factor. After the onset of the HAPI, all six patients were classified as having moderate or high PI risk.

The validity of the PPI Tool was examined by comparing it with the most studied PI risk assessment scale, the Braden Scale. A moderate correlation was found between the scales. In addition, we analysed how well the averages of the Braden Scale scores are divided into PPI Tool's risk classes. The analysis found a significant association that is the patient was classified to be in the same risk class with both measures. A moderate correlation and the statistically significant distribution of the Braden Scale scores into the PPI Tool classes supports the PPI Tool to be reliable. According to this study, the three-category PPI Tool, based on assessment of mobility, skin condition and sensory perceptions is sufficient to identify PI risk patients. These risk factors are the same as the major risk factors identified by EPUAP/NPIAP/PPPIA (2019). The PPI Tool supports nursing staff’s clinical decision-making. Further, the PPI Tool is a part of the Prevent PI Protocol that guides the use of preventive interventions. The short tool is also quick to use, which reduces the risk of not doing a risk assessment in a hurry.

In current health care, it is more demanding to care for the aging population with shorter hospital stays. Nurses should be able to effectively evaluate several nursing care–related concerns, such as the risk of PIs and falls. It is important to assess whether the same result can be achieved with a shorter tool. According to Källman and Lindgren [10], a risk assessment tool should be easy to use so that being in a hurry is not the preventing factor in patients’ PI risk assessment.

The PPI Tool is widely used in the study organization in different environments. This study targeted in-patients in selected wards, and only a very small number of study patients developed a PI. In the future, more research in other nursing environments is needed with larger populations so that the study data contains enough PIs for analysis. In addition, to ensure validity it is still important to compare the PPI Tool with other much-studied tools.

### Limitations

The study presents some limitations that need to be considered. The study population is likely to have been selected because participation required the consent of the participant. During the study, only ten patients developed a HAPI, which affects the reliable analysis of the predictive power of the PPI Tool. In addition, there were only a few patients at high PI risk. In other studies, in acute care, a higher portion of patients were at PI risk than in this study. Incomplete patient records also affected the study. For several participants, their weight, height and malnutrition risk, for example, were missing in their EPRs. Thus, these variables could not be used in the analysis. Additionally, the data did not include information on the use of preventive interventions, so it was not possible to compare whether interventions are implemented more systematically using the PPI Tool than using the Barden Scale. In addition, though nurses were informed not to exchange answers or diffuse information between the PPI Tool and Braden Scale assessments, we have no guarantee that they followed this instruction.

## Conclusions

The aim of this study was to evaluate the validity of the PPI Tool in acute care. The PPI Tool may be useful for identifying the PI risk in adult patients in acute care, in surgical, medical and neurological in-patient wards. The higher PI risk class was related to older age, longer hospital stays, female gender, elective admission to hospital and having a surgical procedure n during treatment. The concurrent validity of the PPI Tool was moderate.

However, more research is required. The PPI Tool needs to be tested in different patient populations and nursing care environments in order to obtain more information about its ability to predict PIs in different care contexts. In addition, interrater reliability and usability need to be examined.

## Relevance to clinical practice

The PPI Risk Assessment Tool was introduced in the study organisation in 2015. In the first phase it was used in wards, but the PPI Tool has been adapted to suit emergency care, ambulance services and children’s wards. In addition, the PPI Tool has modified versions for operating rooms and intensive care units.

PI prevention is an important area of quality nursing care. Identifying the patients’ PI risk is an essential part of preventing PIs in every nursing environment. The PPI Tool is quick to use, so it can contribute to a more systematic assessment of the patients’ PI risk. A reliable risk assessment guides the identification of risk patients to whom preventive interventions can be targeted.

### What does this paper contribute to the wider global clinical community?

- Hospital patients are older and need more care, and at the same time, hospital stays have shortened. Nurses should be able to effectively evaluate several nursing care–related concerns, such as the risk of PIs and falls, in a short time. This requires quick and easy to use assessment tools. New reliable tools are needed for clinical assessments in changing environments.
- The study produced evidence of the validity of the new PPI Tool, which takes into consideration three risk factors: mobility, skin condition and poor sensory perception.
- The results indicate that the PPI Tool may be useful for identifying the adult PI risk patients in acute care, particularly in surgical, medical, and neurological in-patient wards. More research is needed in order to confirm the validity in other patient populations and care environments.

## Supplementary information

**Additional file 1**

## Data Availability

The datasets generated and / or analyzed during the current study are not publicly available due to privacy or ethical restrictions but are available from the corresponding author on reasonable request and with permission of the hospital authorities. Research data are not shared.

## Abbreviations

AUC: area under the roc curve
CI: confidence interval
EPR: electronic patient record
HUS: Helsinki University Hospital
LOS: length of stay
PI: Pressure injure
PPI Tool: Prevent Pressure Injury Risk Assessment Tool
OR: odds ratio
ROC: receiver operating characteristic
RR: relative risk
