# Supplementary material for: Validation of the Helsinki University Hospital prevent pressure Injury Risk Assessment Tool: a prospective observational study
Source: BMC Nurs. 2022 Jan 17;21:18. doi: 10.1186/s12912-021-00799-6 (PMC8762808; doi:10.1186/s12912-021-00799-6)
Supplement: Supplementary file 1 — Additional file 1 [file 12912_2021_799_MOESM1_ESM.pdf]

PREVENTING PRESSURE ULCERS

Assess the patient's risk of pressure ulcers within 2–4 hours of admittance or at least within 8 hours. Reassess the risk every time the patient’s condition changes, but at least once a week.

HUS

1 Assess the risk according to mobility and skin condition

Very limited mobility or an existing pressure ulcer	Limited mobility or fragile skin or loss of sensation	No limitations in mobility and healthy skin
**HIGH RISK**	**MODERATE RISK**	**LOW RISK**
Dynamic, alternating pressure mattress or a high-risk seat cushion	At least a gel memory foam mattress or seat cushion	New assessment when condition changes or at least once a week

2 Check the skin condition

- **Check** the skin especially on bony prominences, and near cannulas, catheters etc.
- **Assess** the skin: dry/moist, changes in colour, tissue elasticity, swelling, abrasions, blisters, and changes in temperature.
- **Assess** pressure ulcers classification and location.
- **Check** the skin at least once per shift (8 h) – more often if the risk is increased.

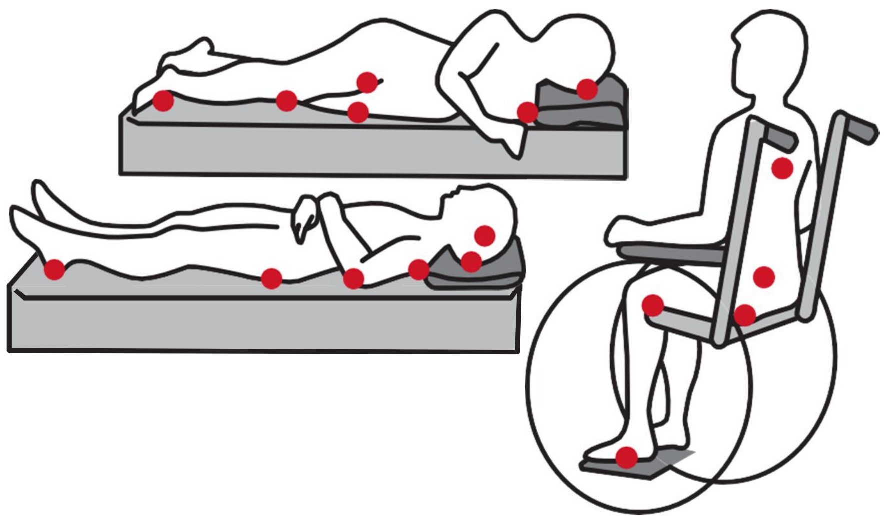

3 Care for the skin and possible incontinence

- **Keep the skin** clean and dry.
- **Change** any moist bed linen and clothes.
- **Apply lotion** on dry skin.
- **Check for incontinence:** Check diapers every 2–3 hours and use protective products on skin.

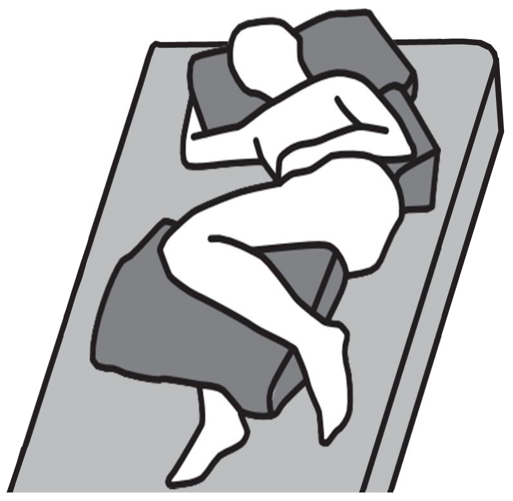

4 Relieve pressure and mobilise

- **Relieve pressure from sitting patients** at least once per hour, from **bed patients** every 2–4 hours, depending on the surface.
- **Use pressure-relieving** positions/repositioning: favour the 30° tilt and mind the heels
- **Prevent** bony prominences from touching each other.
- **Keep the head of the bed** at the lowest possible level, as determined by the patient's condition.
- **Avoid** friction when lifting and moving the patient.
- **Instruct and motivate** the patient to move independently.

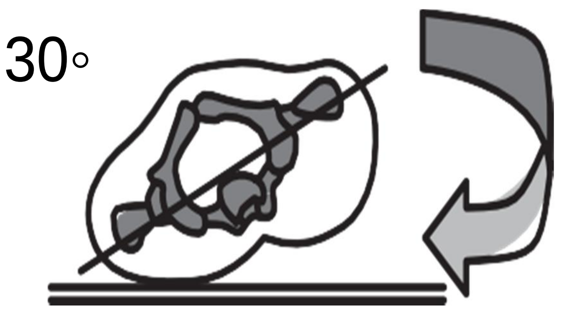

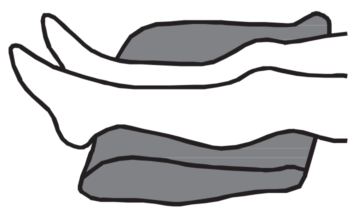

5 Assess the risk of malnutrition

- **Follow** the organisation’s best practice method for assessing the risk of malnutrition.
- **Take care** of nourishment according to the nutrition instructions.

6 Documentation

- **Document** the class of the risk, classification and location of the pressure ulcer, prevention and treatment plan, and all the performed procedures.

HUS, Hospital District of Helsinki and Uusimaa, 2014
